# The Physiological and Molecular Mechanisms of Exogenous Melatonin Promote the Seed Germination of Maize (*Zea mays* L.) under Salt Stress

**DOI:** 10.3390/plants13152142

**Published:** 2024-08-02

**Authors:** Jiajie Wang, Di Yan, Rui Liu, Ting Wang, Yijia Lian, Zhenzong Lu, Yue Hong, Ye Wang, Runzhi Li

**Affiliations:** 1 College of Plant Science and Technology, Beijing University of Agriculture, Beijing 102206, China; jiajie.wang@bua.edu.cn (J.W.); di.yan@bua.edu.cn (D.Y.); rui.liu@bua.edu.cn (R.L.); ting.wang@bua.edu.cn (T.W.); yijia.lian@bua.edu.cn (Y.L.); zhenzong.lu@bua.edu.cn (Z.L.); yue.hong@bua.edu.cn (Y.H.); ye.wang@bua.edu.cn (Y.W.); 2 Beijing Key Laboratory for Agricultural Application and New Technique, Beijing 102206, China

**Keywords:** maize, melatonin, salt stress, seed germination, transcriptome analysis

## Abstract

Salt stress caused by high concentrations of Na^+^ and Cl^-^ in soil is one of the most important abiotic stresses in agricultural production, which seriously affects grain yield. The alleviation of salt stress through the application of exogenous substances is important for grain production. Melatonin (MT, N-acetyl-5-methoxytryptamine) is an indole-like small molecule that can effectively alleviate the damage caused by adversity stress on crops. Current studies have mainly focused on the effects of MT on the physiology and biochemistry of crops at the seedling stage, with fewer studies on the gene regulatory mechanisms of crops at the germination stage. The aim of this study was to explain the mechanism of MT-induced salt tolerance at physiological, biochemical, and molecular levels and to provide a theoretical basis for the resolution of MT-mediated regulatory mechanisms of plant adaptation to salt stress. In this study, we investigated the germination, physiology, and transcript levels of maize seeds, analyzed the relevant differentially expressed genes (DEGs), and examined salt tolerance-related pathways. The results showed that MT could increase the seed germination rate by 14.28–19.04%, improve seed antioxidant enzyme activities (average increase of 11.61%), and reduce reactive oxygen species accumulation and membrane oxidative damage. In addition, MT was involved in regulating the changes of endogenous hormones during the germination of maize seeds under salt stress. Transcriptome results showed that MT affected the activity of antioxidant enzymes, response to stress, and seed germination-related genes in maize seeds under salt stress and regulated the expression of genes related to starch and sucrose metabolism and phytohormone signal transduction pathways. Taken together, the results indicate that exogenous MT can affect the expression of stress response-related genes in salt-stressed maize seeds, enhance the antioxidant capacity of the seeds, reduce the damage induced by salt stress, and thus promote the germination of maize seeds under salt stress. The results provide a theoretical basis for the MT-mediated regulatory mechanism of plant adaptation to salt stress and screen potential candidate genes for molecular breeding of salt-tolerant maize.

## 1. Introduction

With the continuous deterioration of the global ecological environment and the emergence of extreme climate, countries around the world are facing an increasingly severe food security crisis [1]. Rising global temperatures and increased evaporation rates of water from the surface layer of arable land lead to salt accumulation, aggravating salinization of arable land, which inhibits seed germination and leads to crop growth inhibition, and reduced survival and yield [2,3,4]. In addition, salt stress induces other secondary stresses in crops, such as ionic toxicity, osmotic stress, and oxidative stress, disrupting intracellular environmental homeostasis and even leading to cell death [5,6,7,8,9,10].

To adapt to high-salt environments, plants need to maintain their ion homeostasis. Ion transporter proteins (e.g., Na^+^ and K^+^ transporter proteins), which control the input and output of ions, are the first line of defense in the cytoplasmic membrane. The Salt Overly Sensitive (SOS) pathway, discovered and characterized by Zhu’s team, is an important component of the stress signaling pathway that regulates ion homeostasis and salt tolerance in plants [11,12]. Meanwhile, plants have evolved many mechanisms to sense and adapt to osmotic stress, including perception and transduction of osmotic stress signals, phytohormone-related regulation and response, intracellular synthesis, and metabolism of osmotic substances [13,14,15,16,17,18,19]. When oxidative stress induces the production of large amounts of reactive oxygen species (ROS), plants increase the activity of their antioxidant enzymes (such as superoxide dismutase, catalase and peroxidase) to effectively improve the ability to scavenge ROS, reduce plasma membrane damage, and improve tolerance to abiotic stress, including drought, high temperature, salinity, and cold [1,20,21]. Pesticides, as plant protectants, can cause pesticide toxicity when used in excess or inappropriately, and the antioxidant enzyme system is effective in scavenging the accumulation of ROS caused by pesticide toxicity [22].

The use of exogenous hormones to improve plant stress resistance under abiotic stress is one of the most used tools today. MT is an indole-like small molecule that is considered a novel phytohormone. Although this view is still controversial, MT has been found to have many biological functions and plays an important role in plant growth and development and stress response processes [23,24,25,26,27,28,29]. The ability of MT to alleviate growth retardation caused by salt stress and to promote seed germination and plant root development was confirmed by applying MT to soybean, wheat, maize, and rice crops [30,31,32,33]. MT can directly and effectively scavenge ROS and reactive nitrogen species (RNS), as well as other free radicals and harmful oxidative molecules, and act as a signaling molecule to enhance the activity of antioxidant enzymes, including superoxide dismutase (SOD), catalase (CAT), and peroxidase (POD) [2,34,35,36,37]. In addition, MT regulates the expression of the *Arabidopsis* K^+^ transporter protein gene (*AKT1*) and Na^+^/H^+^ reverse transporter protein gene (*NHX1*) to maintain ion homeostasis and mitigate plant damage from salt stress [38,39]. MT is involved in multiple signaling pathways and interacts with several phytohormones to regulate plant growth and improve stress tolerance. MT has been reported to interact with abscisic acid (ABA) and gibberellin (GA) to affect the biosynthesis and catabolism of ABA and GA, disrupt ABA/GAs, and promote seed germination under salt stress [30,36,40]. MT activates the MAPK cascade and enhances tolerance to salt stress and immunity to pathogens in *Arabidopsis* [41], tobacco [42], Bermuda grass, and other plants [43]. Exogenous MT upregulates the expression of key genes in the GA synthesis pathway, such as *GA*_20_*ox* and *GA*_3_*ox*, leading to an increase in GA content, and regulates the crop response to GA by modulating DALLE proteins in the GA signaling pathway under salt stress [30,36]. However, most of the current studies on MT are at the physiological level, and the molecular mechanism of its action is still not clearly resolved.

Maize (*Zea mays* L.) is one of the most important and widely grown crops in the world. In addition to being a grain, maize is an important source of feed and industrial raw materials with high economic value [44]. At the same time, maize cultivation is being threatened by a variety of environmental problems, such as high and low temperatures, drought, salinity, and flooding. Therefore, how to improve the stress tolerance of maize seeds has become a research priority. Despite several studies on MT in crop stress tolerance, the molecular mechanisms during maize seed germination after MT application under salt stress are still unclear. In this study, we investigated the germination indexes, physiological indexes, endogenous hormone contents, and transcript levels of maize seeds at different germination stages under salt stress to gain insights into the exogenous MT-mediated physiological mechanisms and gene regulatory networks of maize seed germination stages in response to salt stress.

## 2. Results

### 2.1. Effect of Exogenous MT on Emergence Traits of Maize under Salt Stress

**Figure 1:**
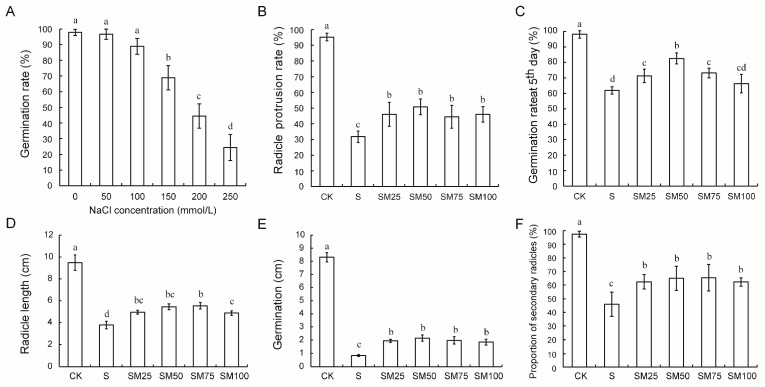
The germination effect of 150 mM NaCl on maize seeds. (**A**) Germination rate of maize seeds at different NaCl concentrations; (**B**) rate of radicle breakthrough of seed coat at the 36th hour; (**C**) germination rate at 5th day; (**D**) radicle length; (**E**) germination length; (**F**) proportion of secondary radicle occurrence. Different letters indicate statistically significant differences (ANOVA with LSD Fisher’s multiple comparison test, *p* < 0.05).

As shown in Figure 1A, the seed germination rate was significantly decreased under NaCl stress of 150 mM compared with the control, and 150 mM NaCl was selected as the salt stress concentration for this experiment, in combination with the sampling needs of the subsequent experiments. Pretreatment with MT significantly increased the germination rate of maize seeds by 7.07–33.33% under NaCl stress conditions, with SM50 treatment having the most significant effect (Figure 1C). In addition, the radicle protrusion rate was promoted by 44.98–59.97% under MT treatment compared to salt stress (Figure 1B). Combined with its radicle protrusion rate, the results showed that MT was able to significantly increase the seed germination rate and alleviate the growth and developmental retardation induced by salt stress (Figure 1B,C). Both radicle length and germination length are important indicators of seed germination. Salt stress inhibits the development of seed radicle and germination (decreased by 60.29% and 89.86%, respectively), but the application of MT alleviated this developmental retardation caused by salt stress (29.24–46.84% and 119.58–155.19% increases, respectively, compared to S treatment) (Figure 1D,E). Notably, MT promoted the development of the secondary radicle of maize seeds under salt stress. The percentage of germinated seeds that developed secondary radicles increased by an average of 38.73% after MT treatment compared to group S (Figure 1F). These results are consistent with observations on seed phenotypes (Figure S1), suggesting that MT is effective in promoting growth and development during seed germination.

### 2.2. Effect of MT on Oxidative Damage and Osmoregulation in Seeds

**Figure 2:**
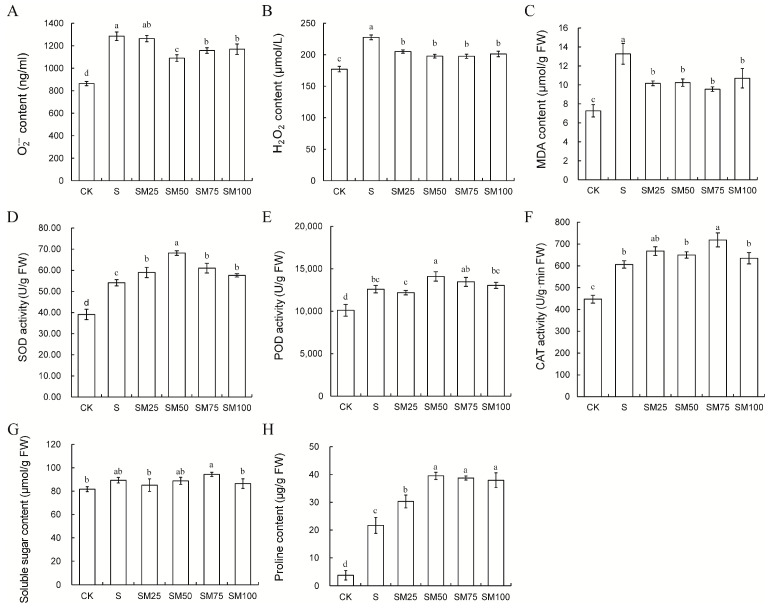
Effect of MT on physiological traits during germination of maize seeds. (**A**) O_2_^−^ content; (**B**) H_2_O_2_ content; (**C**) MDA content; (**D**) SOD activity; (**E**) POD activity; (**F**) CAT activity; (**G**) soluble sugar content; and (**H**) proline content. Different letters indicate statistically significant differences (ANOVA with LSD Fisher’s multiple comparison test, *p* < 0.05).

Under salt stress induction, ROS content and MDA, a product of oxidative damage to membrane plasma, were significantly higher in maize seeds compared with CK. In contrast, O_2_^−^, H_2_O_2_, and MDA contents were significantly reduced after pretreatment with MT (Figure 2A–C).

ROS stimulated the plants to increase antioxidant enzyme activities, in which SOD activity was elevated by 38.52%, POD and CAT activities by 24.48% and 35.47%, respectively, in the S group compared to the CK group (Figure 2D–F). However, the antioxidant enzyme activities of maize seeds were significantly higher under SM treatment than S treatment. The SOD and POD activities of maize seeds reached their maximum at SM50 treatment, which increased by 25.9% and 11.88%, respectively, compared with S group (Figure 2D,E). And among all SM groups, only the SM75 group significantly increased CAT activity by 18.56% relative to S group (Figure 2F).

The content of soluble sugars showed that salt stress, as well as MT treatment, had little effect on the content of soluble sugars in maize seeds (Figure 2G). This may be caused by the fact that at this time the seeds were in the period of germination and the nutrients in the endosperm were being converted into soluble sugars. However, the determination of proline content showed that salt stress caused a large accumulation of proline in maize seeds (473.26% increase compared with the CK group), and MT pretreatment further significantly increased the proline content under salt stress, where SM50 increased by 82.44% (Figure 2H).

Considering the results of seed germination, ROS content, and the activities of various antioxidant enzymes, the MT concentration of 50 μM was finally selected as the optimal concentration for subsequent experimental analyses.

### 2.3. Effect of MT on Endogenous Hormone Levels

**Figure 3:**
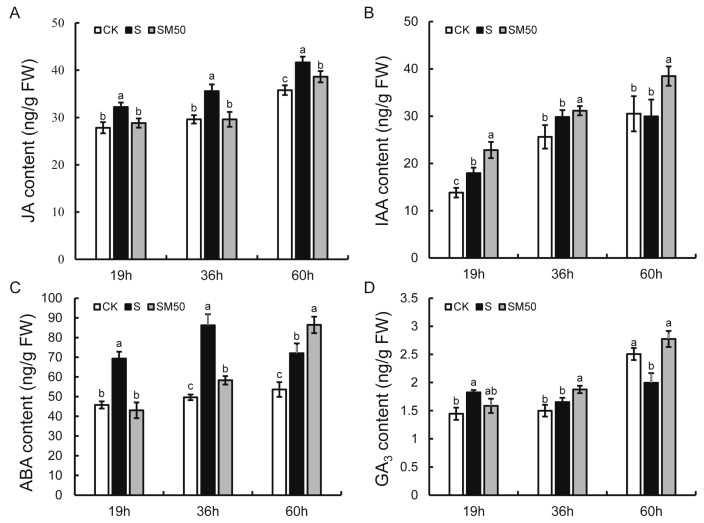
Hormone contents under different germination stages of different treatments. (**A**) JA content; (**B**) IAA content; (**C**) ABA content; (**D**) GA_3_ content. Different letters indicate statistically significant differences (ANOVA with LSD Fisher’s multiple comparison test, *p* < 0.05).

To investigate the mechanism of the MT-induced germination of maize seeds under salt stress, we measured the contents of several phytohormones at three stages: seed imbibition (19 h), radicle protrusion (36 h), and germination (60 h). Under normal conditions, the contents of all four phytohormones, GA_3_, ABA, IAA, and JA, increased during seed germination (Figure 3). Compared with CK, under salt stress conditions, the contents of JA and ABA were significantly increased at all three germination stages, with increases of 15.75–20.15% and 34.39–73.82%, respectively (Figure 3A,C). The contents of GA_3_ and IAA increased by 26.07% at 19 h and 29.73% at 36 h and decreased by 20.42% and 1.76% at 60 h, respectively (Figure 3B,D). In the MT pre-treated samples, the content of JA was significantly reduced (7.24–16.78%) at all three germination stages compared to untreated ones, whereas the opposite was true for IAA (increased by 4.38–28.37%) (Figure 3A,B). The content of ABA and GA_3_ increased by 20.06% and 39.14% at 60 h and decreased by 37.94% and 13.05% at 19 h, respectively. But, at 36 h ABA decreased by 32.44%, while GA_3_ increased by 13.52% (Figure 3C,D).

### 2.4. Transcriptome Data Quality Assessment and Analysis of DEGs

**Figure 4:**
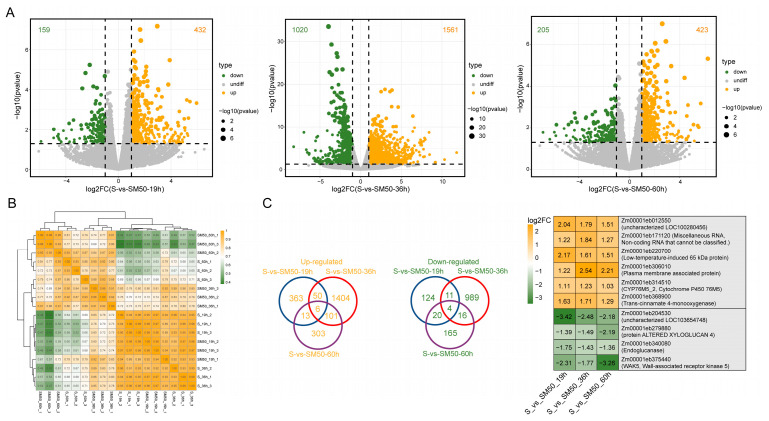
Effect of MT on gene expression of maize seeds at different germination stages under salt stress. (**A**) Volcano plot of differential genes; (**B**) correlation analysis of all samples and sample clustering; (**C**) Venn plot of up- and down-regulated differential genes and heat map of differential expression fold change of overlapping genes.

To reveal the transcriptional mechanism by which MT promotes maize seed germination under salt stress, transcriptome sequencing was performed for S-vs-SM50 at three different stages (19 h, 36 h, and 60 h) during seed germination. The quality assessment of the sequencing data of all samples was shown in Table S2, and a total of 851,402,640 Clean Reads were obtained from the transcriptome sequencing. The average Q20 and Q30 of all samples were 97.03% and 92.45%, respectively, while the average GC content was 55.12%. The expression patterns of the four genes used for qRT-PCR analysis followed the same trend as that of the transcriptome data (Table S1, Figure S2), indicating that the sequencing results were good for subsequent analysis. The results of sample correlation analysis showed that the three biological replicates of each treatment were well clustered together, indicating good reproducibility of the samples within the group and better reflecting the fact that the MT pretreatment has a regulatory effect on gene transcription in maize seeds at the germination stage (Figure 4B). Interestingly, samples from the S and SM50 groups clustered together at 19 h and 60 h but were clearly distinguished at 36 h, which was during the radicle protrusion stage, suggesting that MT affects gene transcription in maize seeds mainly at that stage (Figure 4B).

A total of 3509 DEGs were identified in the comparison of S-vs-SM50 at the three stages of germination, and 432 up-regulated genes and 159 down-regulated genes at 19 h and 423 up-regulated genes and 205 down-regulated genes at 60 h were observed. The highest number of DEGs was identified at 36 h, with 1561 up-regulated and 1020 down-regulated genes (Figure 4A). This result likewise indicates that MT has a greater effect on gene transcript expression during the radicle protrusion stage. To further understand the effect of MT on DEGs at different germination stages in S-vs-SM50, a Venn diagram analysis was performed on the up-regulated DEGs and down-regulated DEGs at the three germination stages, respectively (Figure 4C). The results showed that a total of six of the up-regulated DEGs were affected by MT and continued to be up-regulated throughout the germination phase, whereas a total of four of the down-regulated DEGs were affected by MT and continued to be down-regulated throughout the germination phase (Figure 4C).

### 2.5. GO and KEGG Enrichment Analysis of DEGs

**Figure 5:**
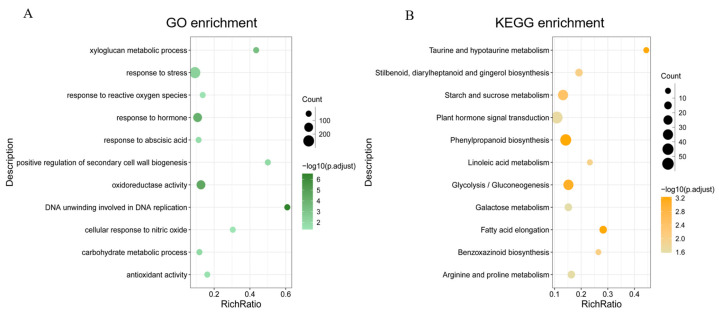
Differentially expressed gene enrichment analysis. (**A**) GO enrichment analysis; (**B**) KEGG enrichment analysis. The vertical axis represents the pathway name, the horizontal axis represents the Rich Ratio, the size of dots in the pathway represents the count of DEGs, and −log10 (p.adjust) is the significance of the enrichment reflected by the color of dots.

To identify the major pathways regulated by MT to promote seed germination, all DEGs were analyzed for GO and KEGG enrichment, and 11 significantly enriched entries and pathways were each selected from the enrichment results for bubble mapping. GO enrichment analysis showed that these DEGs were mainly enriched in the xyloglucan metabolic process, response to stress, response to reactive oxygen species, response to hormones, response to abscisic acid, positive regulation of secondary cell wall biogenesis, oxidoreductase activity, DNA unwinding involved in DNA replication, cellular response to nitric oxide, the carbohydrate metabolic process, and antioxidant activity (Figure 5A). Unsurprisingly, MT further affects seed germination under stress mainly by regulating the expression of genes involved in stress response, hormone response, antioxidant capacity, and energy substance metabolism-related genes. KEGG enrichment analysis showed that MT-affected DGEs were mainly enriched in phenylpropane-related synthetic pathways such as phenylpropanoid biosynthesis and flavonoid biosynthesis. DEGs were similarly enriched in lipid-related pathways such as linoleic acid metabolism and fatty-acid elongation and glycan-related pathways such as glycolysis/gluconeogenesis and starch and sucrose metabolism (Figure 5B). In addition, the pathways of plant hormone signal transduction and benzoxazinoid biosynthesis were also enriched by DEGs (Figure 5B).

### 2.6. Time Series Analysis of Expression Patterns of DEGs

**Figure 6:**
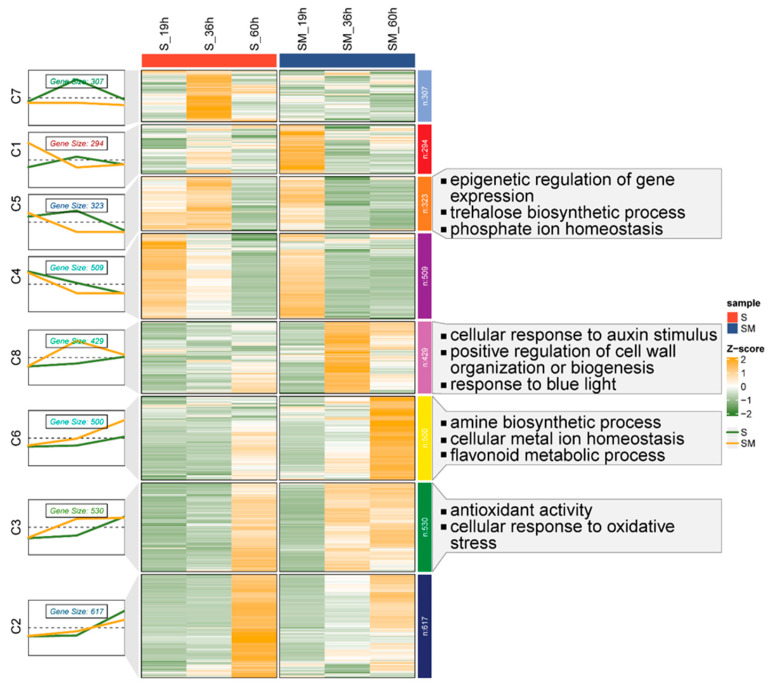
Time series analysis of differentially expressed genes. Heat map of expression pattern clustering and GO enrichment analysis of DEGs in SM vs. S comparison. The folded line graph represented the Mfuzz clustered expression pattern of DEGs.

To investigate the expression and function of DEGs at different germination stages, time series analysis of all DEGs was performed to further analyze their expression patterns. As shown, all DEGs were clustered into eight clusters according to their expression patterns (Figure 6). Based on the expression patterns of the genes in these eight clusters, as well as the expression heat map, C5, C8, C6, and C3, which were more in line with expectations, were selected for further analysis. These gene clusters clustered 323, 429, 500, and 530 DEGs, respectively. The genes clustered in C5 were similarly expressed at 19 h and 60 h, but the expression of MT-pretreated DEGs was significantly higher than that of the S group at 36 h, whereas the expression pattern of DEGs in C8 and C3 was the opposite of that of C5 (Figure 6). Expression pattern plots of C6 showed that the expression of such DEGs in the S and SM50 groups was similar in the early stage (19 h), but the expression of these genes gradually increased as the germination process progressed, and MT pretreatment further increased their expression (Figure 6).

GO enrichment analyses of DEGs in each of these gene clusters showed that DEGs in C5 were mainly involved in epigenetic regulation of gene expression and trehalose biosynthetic process. This suggests that MT may protect normal gene expression from salt stress by suppressing the epigenetic regulation of gene expression and increase energy supply and promote seed radicle development by inhibiting the conversion of glucose to trehalose (Figure 6). DEGs in C6 are mainly associated with the amine biosynthetic process, cellular metal ion homeostasis, and the flavonoid metabolic process (Figure 6). This suggests that MT has a positive effect on ion homeostasis in cells. The expression patterns of DEGs in C8 and C3 were similar and GO enrichment analysis showed that they were involved in the cellular response to auxin stimulus, positive regulation of cell wall organization or biogenesis, antioxidant activity, and cellular response to oxidative stress, respectively. This further suggests that MT has a greater impact on seeds during the radicle protrusion stage, increasing their ability to scavenge reactive oxygen species and their response to phytohormones, which in turn has the effect of promoting seed germination (Figure 6).

### 2.7. Gene Set Enrichment Analysis of S-vs-SM50 in Different Germination Stages

In order to reveal the unique roles played by MT in the three key stages of germination, GO and KEGG gene set enrichment analyses were performed for all genes in the three germination stages.

**Figure 7:**
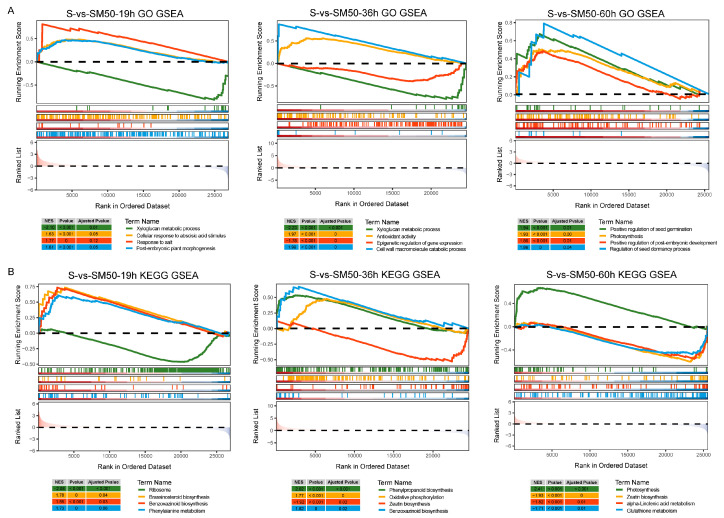
Gene set enrichment analysis of S-vs-SM50 in different germination stages. (**A**) GO gene set enrichment analysis; (**B**) KEGG gene set enrichment analysis.

GO gene set enrichment analysis (Figure 7A) showed that genes related to the xyloglucan metabolic process were down-regulated in maize seeds and genes related to cellular response to abscisic acid stimulus, response to salt, and post-embryonic plant morphogenesis were up-regulated in maize seeds at the time of seed imbibition stage (19 h). This suggests that MT at this time enhances seed response to ABA and salt in response to salt stress and inhibits xyloglucan metabolism to promote seed embryo development. When the seeds were in the radicle protrusion stage (36 h), the genes related to xyloglucan metabolic process and epigenetic regulation of gene expression were down-regulated, and the genes related to antioxidant activity and the cell wall macromolecule catabolic process were up-regulated. This is consistent with the results obtained from the time series analysis. When the seeds were in the germination stage (60 h), the GO entries showed that MT mainly up-regulated positive regulation of seed germination, positive regulation of post-embryonic development, photosynthesis, and regulation of the seed dormancy process. This demonstrated that MT promoted maize seed germination under salt stress by up-regulating seed germination-related genes.

The results of the KEGG gene set enrichment analysis (Figure 7B) showed in the MT affected genome set an overall down-regulation of ribosome-related genes, and an overall up-regulation of genes related to benzoxazinoid biosynthesis, brassinosteroid biosynthesis, and phenylalanine metabolism in the seed imbibition stage (19 h). Among the gene sets at the seed radicle protrusion stage (36 h), there was an overall down-regulation of genes related to zeatin biosynthesis, and an overall up-regulation of genes related to phenylpropanoid biosynthesis, oxidative phosphorylation, and benzoxazinoid biosynthesis. It is noteworthy that the biosynthetic pathways of benzoxazoles were up-regulated by MT, both in the DEG enrichment analysis and in the gene set enrichment analysis, implying that MT has a positive impact on insect resistance and the antimicrobial and chemosensory effects of crops, in addition to the enhancement of crop resistance and the promotion of seed germination. The genes related to photosynthesis were up-regulated, while the genes related to zeatin biosynthesis, alpha-linolenic acid metabolism, and glutathione metabolism were down-regulated at the seed germination stage (60 h). At this time, MT inhibited the expression of genes related to glutathione metabolism and increased its accumulation, which helped the seeds to improve salt tolerance.

### 2.8. Effect of MT on Phytohormone Signal Transduction Pathways during Seed Germination under Salt Stress

**Figure 8:**
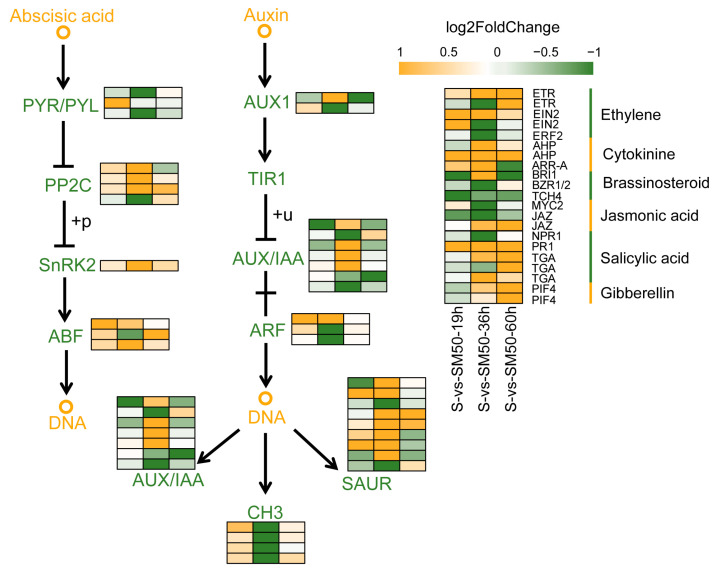
Effect of MT during germination on phytohormone signaling pathways in maize seeds under salt stress.

Based on the KEGG enrichment analysis of DEGs and the results of the determination of endogenous plant hormone contents, this study speculates that MT largely influences the germination process of seeds under adversity by interacting with other plant hormones. Throughout the three key stages of the germination process, a total of 57 DEGs belonged to genes related to the phytohormone signal transduction pathway, of which the IAA pathway had the highest number of DEGs with 25, followed by the ABA pathway with 11 DEGs (Figure 8). Both the SA pathway and the ETH pathway had five DEGs, CTK, BR, and JA had three DEGs each, and GA had only two DEGs (Figure 8). Based on the differential gene expression and pathway maps, it can be seen that although the expression of the negative regulator *ZmPP2C* in the ABA pathway was up-regulated, the expression of its downstream genes, *ZmSnRK2* and the ABA response element binding factor *ZmABF*, were similarly up-regulated (Figure 8). In the IAA pathway, partial up-regulation and partial down-regulation of *ZmAUX/IAA* promoted *ZmSUAR* expression overall, whereas *ZmCH3* expression was up-regulated at both 19 h and 60 h and was significantly down-regulated only at 36 h (Figure 8). Among the remaining hormone pathways, the ETH, CTK, SA, and GA pathways were overall positively regulated by MT, whereas the BR and JA pathways were negatively regulated by MT (Figure 8). This suggests that MT is involved in regulating the signal transduction of multiple phytohormones, thus affecting a variety of biological processes and that MT further regulates seed salt tolerance and germination processes mainly by regulating the expression of genes related to the IAA and ABA signal transduction pathways.

### 2.9. Effect of MT on Starch and Sucrose Metabolic Pathways during Seed Germination under Salt Stress

**Figure 9:**
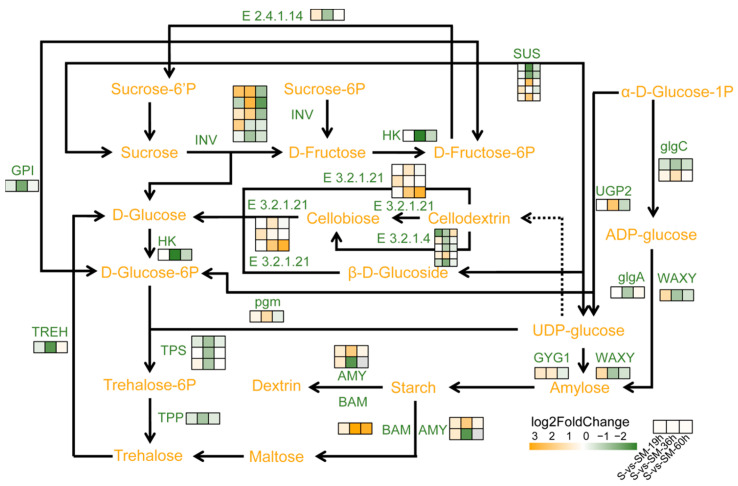
Effect of MT during germination on starch and sucrose metabolism in maize seeds under salt stress.

MT regulated a total of 39 DEGs in the starch and sucrose metabolic pathway. Analysis of the starch and sucrose metabolic pathway showed that MT up-regulated starch and sucrose catabolic genes, such as *ZmINV,* encoding β-fructofuranosidase, and *ZmSUS,* encoding sucrose synthetase, which are associated with the catabolism of sucrose, and *ZmBAM* and *ZmAMY,* encoding α-amylase, which are associated with the catabolism of starch (Figure 9). In addition, MT up-regulated genes related to the synthesis of D-glucose, such as *ZmE3.2.1.21,* encoding β-glucosidase, and repressed genes related to the catabolism of D-glucose, such as *ZmHK,* encoding hexokinase (Figure 9). Meanwhile, MT treatment reduced the expression of genes related to the synthesis of trehalose, such as *ZmTPS,* encoding trehalose-6-phosphate synthase, and *ZmTPP,* encoding trehalose-6-phosphate phosphatase, compared with that of group S (Figure 9). In conclusion, MT pretreatment promoted the decomposition of starch and sucrose and the synthesis of glucose in maize seeds under salt stress, providing sufficient energy for seed germination.

### 2.10. Effect of MT on Transcription Factors during Seed Germination under Salt Stress

**Figure 10:**
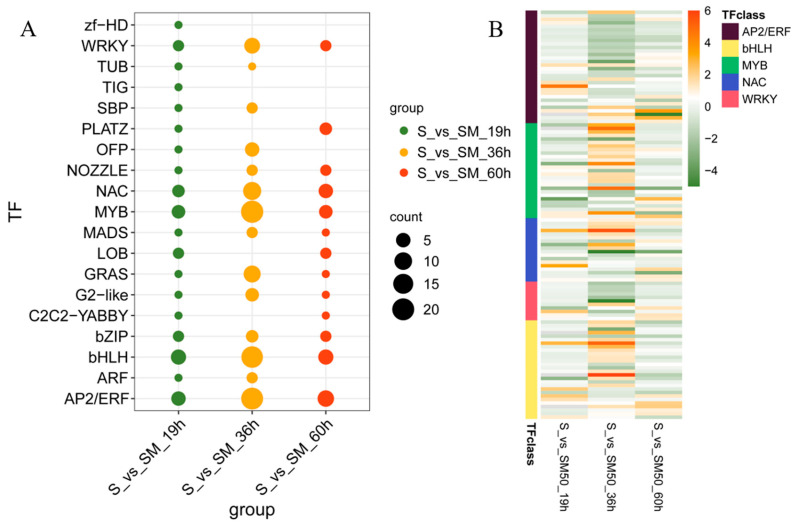
Effect of MT during germination on gene expression of transduction factors in maize seeds under salt stress. (**A**) Classifications and counts of TFs in the S-vs-SM comparisons at different times. (**B**) Heatmaps of the top 5 TFs families in the S-vs-SM comparisons at different times.

In the plant hormone response and signal transduction pathways, there are many DEGs of transcription factors involved in the response to salt stress, MT, and the regulation of seed germination, such as ABF in the ABA pathway, ARF in the IAA pathway, PIF in the GA pathway, and JAZ and MYC2 in the JA pathway, among others. In addition to their involvement in hormone signaling, some transcription factors are also associated with plant stress tolerance. In this study, 19 transcription factor family members were identified in all DEGs. Regardless of the germination stage, these transcription factor genes were mainly classified into the AP2/ERF, bHLH, MYB, NAC, and WRKY families (Figure 10A). The heatmap shows the expression differences of TFs in the comparison of S-vs-SM50, which are mainly regulated by MT at 36 h (Figure 10B). In addition, MT-regulated genes of the NAC, MYB, and WRKY families were associated with salt tolerance in plants, which may be one of the ways in which MT improves salt tolerance in maize seeds and promotes seed germination.

## 3. Discussion

### 3.1. MT Alleviates Salt Stress-Induced Oxidative Damage by Enhancing the Ability to Scavenge ROS

Excessive accumulation of salt in the soil inhibits seed germination. In practical agricultural production, plant hormones and growth regulators, such as GA, JA, SA, IAA, CTK, ABA, and their functional analogues, are commonly used to promote seed germination, reduce insect pests and diseases, and mitigate damage caused by environmental stresses [31,32,45,46,47,48,49,50,51]. MT is a new indole hormone, which is also widely used to alleviate developmental retardation and oxidative damage caused by salt stress and to improve plant stress tolerance [46]. The results of this study showed that salt stress inhibited maize seed germination, but exogenous MT pretreatment effectively alleviated salt stress-induced developmental retardation, promoted seed germination, and significantly promoted lateral rooting (Figure 1). Changes in MDA content represented the degree of oxidative damage to the plant plasma membrane caused by salt stress. In this study, salt stress led to an increase in MDA content in maize seeds, but MDA content was significantly lower in MT treatment compared with no MT treatment. This indicated that MT mitigated the salt stress oxidative damage to maize seeds (Figure 2C).

O_2_^−^ and H_2_O_2_ in ROS can act as signaling molecules to activate plant responses to unfavorable environmental conditions. However, high-salinity-induced over-accumulation of O_2_^−^ and H_2_O_2_ can seriously threaten the membrane structure of plant cells [52]. MT, an antioxidant, is capable of scavenging excessive accumulation of ROS directly through a cascade reaction [53]. According to the results of this study, O_2_^−^ and H_2_O_2_ were maintained at low levels in MT-treated maize seeds under salt stress (Figure 2A,B). In addition, MT has been reported to be able to regulate ROS homeostasis by increasing the activity of antioxidant enzymes [46,54]. The results of this study showed that MT increased the activities of SOD, CAT, and POD in maize seeds under salt stress (Figure 4). The same results were obtained when exogenous MT was applied to wheat, rice, soybean, and tomato under salt stress [55,56,57,58]. Based on these results, the present study suggests that MT promotes seed germination by directly or indirectly participating in the regulation of ROS homeostasis in plants under stress to avoid overreaction to stress.

### 3.2. MT Improves Seed Germination under Salt Stress by Regulating Phytohormone Levels and Phytohormone Signal Transduction Pathways

Endogenous hormones play a crucial role in seed germination [59]. In addition to enhancing the antioxidant capacity of plants under salt stress, MT is also involved as a signaling molecule in regulating hormone signal transduction and activating the stress response of plants, thereby improving stress tolerance [35]. As shown in previous studies, phytohormones (e.g., ABA, IAA, BR, CTK, JA, SA, and ETH) enable plants to regulate their growth and development in response to the growing environment and help them adapt to unfavorable conditions [60]. For example, ABA plays a role in all adversity stresses and enhances stress tolerance in wheat [61], maize [62], and soybean [63] through exogenous ABA. IAA and JA play crucial roles as endogenous signaling molecules in both plant growth and response to adversity [64,65].

In the ABA signal transduction pathway, PYR/PYL/RCAR, as ABA receptors, phosphorylate the ABA response element binding factor ABF by activating SnRK2 and initiate the expression of genes downstream of the ABA response pathway by inhibiting the activity of the ABA negative regulator PP2C [66]. In this study, MT significantly reduced ABA levels in seeds and suppressed the response to ABA signaling. The transcriptome results showed that *ZmPP2C* was significantly up-regulated in SM50 treatment, but *ZmPYR/PYL* was significantly down-regulated (Figure 3 and Figure 8). Interestingly, *ZmSnRK2* and *ZmABF*, which should have been repressed due to the up-regulation of *ZmPP2C*, were both up-regulated in the SM50 treatment (Figure 8). In view of these results, this study speculated that MT promotes seed germination under salt stress by reducing ABA signaling to avoid inhibition of seed germination due to accumulation of ABA and over-responsiveness to ABA, while activating stress-responsive genes through other pathways to achieve a balance between plant growth and stress tolerance. Previous studies have shown similar results; e.g., MT alleviated salt stress damage to wheat and cucumber seeds and promoted seed germination by reducing ABA biosynthesis and signaling pathways [30,36]. In this study, MT increased IAA levels at three different stages of seed germination (Figure 3). Whereas in the IAA signaling pathway some *ZmAUX/IAA* was partially up-regulated and partially down-regulated and in general promoted the expression of *ZmSUAR*, *ZmCH3* was up-regulated at both 19 and 60 h and was significantly down-regulated only at 36 h (Figure 8). In the IAA pathway, IAA is sensed by TIR1 in the E3 ubiquitin–protein ligase SCF complex, and the complex then ubiquitinates AUX/IAA and degrades it via the 26 S proteasome, releasing ARF to activate downstream response genes [60]. The results of the present study were similar to those of MT applied to *Arabidopsis* and cucumber, suggesting that MT may improve germination of maize seeds under salt stress by activating IAA downstream response genes [67,68].

### 3.3. MT Promotes Seed Germination under Salt Stress by Regulating Starch and Sucrose Metabolic Pathways during Seed Germination

Sugars function as structural materials for seed development and energy supply during germination to maintain osmotic pressure in plants to resist salt stress [69]. For example, carbohydrate metabolites (e.g., sucrose, D-glucose, and D-fructose) are up-regulated during the later stages of salt stress in watermelon to improve salt tolerance [70]. Starch is the end product of plant photosynthesis and is one of the sources of energy for plants. When subjected to extreme environmental stimuli, plants often increase sugar accumulation by regulating starch catabolic pathways to improve their salt tolerance [71,72,73]. In this study, MT upregulated *ZmAMY*, a gene encoding α-amylase, and *ZmBAM*, a gene encoding β-amylase, to promote starch catabolism (Figure 9). Both sucrose and trehalose are key metabolites in starch and sucrose metabolism. Among them, sucrose can be further degraded to UPD glucose, a precursor for the synthesis of trehalose 6-phosphate, and both trehalose and sucrose have been found to improve salt tolerance in plants [74,75,76]. However, transcriptome analysis in this study showed that the transcript levels of trehalose-6-phosphatase (TPP), responsible for the synthesis of trehalose, and trehalose-6-synthase (TPS), responsible for the synthesis of trehalose 6-phosphate, were lower in the SM50 treatment than in the S treatment, while the expression of genes coding for enzymes responsible for the degradation of sucrose (e.g., β-fructofuranosidase, INV, and sucrose synthetase, SUS) was up-regulated, which suggests that the MT promoted the breakdown of sugar in maize seeds under salt stress and inhibited the synthesis of trehalose, which promoted the accumulation of D-glucose (Figure 9). This result seems to be in conflict with previous results [77], and in this study, we speculate that MT promotes sucrose and starch catabolism and increases D-glucose content in order to increase energy supply and promote seed germination while improving the salt tolerance of seeds through other pathways such as increasing antioxidant capacity and up-regulation of stress-responsive genes.

### 3.4. MT Regulates the Expression of Different Functional Genes at Different Seed Germination Stages to Promote Seed Germination under Salt Stress

Seed germination is a complex and unique physiological process that initiates crop growth and development [78]. How to improve the seedling establishment rate of crops under stress has been the focus of attention, but most of the research has stayed on physiology and phenotype and has not carried out in-depth studies on seed germination at the molecular level [79]. Seed imbibition, radicle protrusion, and emergence are three important stages in the seed germination process, but the effects of MT on these three germination stages have not been reported yet. The results of this study showed that MT affects the expression of genes with different functions at different stages of seed germination, thus improving salt tolerance and promoting seed germination throughout the germination period. For example, at the seed imbibition stage (19 h), MT mainly regulated the formation of cell walls and the response of ABA and salt stress during embryo development, which was the same function as in the wheat seed [30].

Just like our previous research on the wheat seed [30], at the radicle protrusion stage (36 h), MT mainly affected the expression of epigenetic regulation of gene expression, antioxidant enzymes, and cell wall formation. At the seed germination stage (60 h), MT affected genes related to the positive regulation of seed germination, photosynthesis, and zeatin biosynthesis (Figure 7). This study demonstrated that MT, as a multifunctional indole hormone, plays an important role in promoting seed germination through a complex regulatory network throughout the germination process under salt stress. However, since the signal transduction pathway of MT is still not well resolved, the question of how MT functions during seed germination needs to be further explored.

## 4. Materials and Methods

### 4.1. Materials and Treatments

The material used in this experiment was the maize inbred line B73, provided by the Seed Science Laboratory, College of Plant Science and Technology, Beijing University of Agriculture. Full, uniformly sized B73 maize seeds with undamaged surfaces were selected and sterilized with a 1% NaClO solution for 10 min and then rinsed three times with deionized water before use.

### 4.2. Salt Stress Concentration Screening

The salt solution concentration gradient was set at 0, 50, 100, 150, 200, 250 mM NaCl. Seeds were soaked in deionized water for 12 h, neatly arranged in germination boxes and placed in a smart light incubator for seven days for seed germination experiments (temperature: 20–25 °C, light: dark incubation for the first three days, and 12 h each of light and darkness for the last four days). Three biological replicates were set up for each treatment, and 30 seeds were placed in each replicate.

### 4.3. Optimal MT Concentration Screening

The treatment of MT-dipped seeds was adopted for the concentration screening experiment. Six treatment groups were set up: control (CK), 150 mM NaCl + 0 μM MT (S), 150 mM NaCl + 25 μM MT (SM25), 150 mM NaCl + 50 μM MT (SM50), 150 mM NaCl + 75 μM MT (SM75), 150 mM NaCl + 100 μM MT (SM100). Four biological replicates were set up for each treatment and 40 seeds were placed in each replicate. Sampling was performed when incubated under salt stress conditions until the seventh day. The samples were snap-frozen with liquid nitrogen and stored in an ultra-low-temperature refrigerator at −80 °C, and three biological replicates were taken for each treatment.

### 4.4. Endogenous Hormone Content and Transcriptome Sequencing

Three treatments were set up: control (CK), salt treatment (S), and salt + MT treatment (SM50). Three biological replicates were set up for each treatment, and each replicate was used with 40 seeds placed in the incubator for germination experiments. The germinated seeds at 19 h, 36 h, and 60 h were sampled for the determination of endogenous plant hormone content and transcriptome sequencing, respectively.

### 4.5. Measurement of Germination and Morphological Indicators

The number of seeds dewed and sprouted and the lateral radicle occurrence were counted regularly every day, and radicle length and germ length were measured at the 7th day. Lateral radicle, i.e., in addition to the main radicle, is the radicle that grows from the upper side of the shield stem at the base of the mesocotyl.

### 4.6. Measurement of Antioxidant Enzyme Activities

The frozen samples were quickly ground to powder in a pre-cooled mortar with liquid nitrogen, and 0.2 g of powder was added to a 2 mL centrifuge tube, and then 1.6 mL of phosphate buffer (pH = 7.8, 0.05 M) was added for extraction and then put into a centrifuge to be centrifuged (4 °C, 10,000 rpm, 20 min). The supernatant was used for the determination of antioxidant enzyme activity and stored in the refrigerator at 4 °C.

The SOD activity was measured by the nitrogen blue tetrazolium (NBT) method [80]. Briefly, 100 μL of crude extract was added to 3.9 mL of the reaction mixture and placed under 4000 Lx intense light for 30 min, then protected from light. The control group was an equal amount of reaction solution. The SOD activity was determined and calculated at 560 nm.

POD activity was measured by guaiacol method [81]. Briefly, 40 μL of crude extract was taken and added to 3 mL of reaction solution at 470 nm to determine the change in absorbance for 1 min. The control was the inactivated enzyme solution after boiling. POD activity was calculated as one unit of enzyme activity per minute increase in absorbance value of 0.01.

The CAT activity was measured by UV spectrophotometric method [81]. Briefly, 50 μL of enzyme solution was added to 3 mL of buffer (PH = 7.8, 0.05 M), and 200 μL of 0.3% H_2_O_2_ was added, shaken quickly, and then the change in absorbance was measured at 240 nm for 1 min. The inactivated enzyme solution after boiling was used as a control to calculate CAT activity.

### 4.7. Measurement of MDA and Soluble Sugar Content

The MDA content was determined by the thiobarbituric acid method 30 by mixing 0.2 g of sample powder with 8 mL of 10% trichloroacetic acid (TCA) and centrifuging (4 °C, 4000 rpm, 10 min). Then, 2 mL of supernatant (water as control) was taken, and 2 mL of 0.6% thiobarbituric acid (TBA) solution was added and mixed well, and the mixture was put in boiling water for 15 min, cooled quickly, and centrifuged. The absorbance values of the supernatant were measured at 450 nm, 532 nm, and 600 nm, and the MDA and soluble sugar contents were calculated.

### 4.8. Reactive Oxygen Content Measurement

The H_2_O_2_ and O_2_^−^ content were determined with reference to the method of Lu et al. [81].

H_2_O_2_ content measurement: 0.2 g of the sample was taken in 4 mL of 0.1% (*w*/*v*) TCA and ground to homogenization and centrifuged at 10,000 rpm for 10 min. The precipitates were then thoroughly mixed to 1 mL with 0.2 mL of ammonia and 0.1 mL of 95% (*v*/*v*) hydrochloric acid solution containing 20% (*v*/*v*) TiC1_4_ and centrifuged (4 °C, 4000 rpm, 10 min). After removing the supernatant, the precipitate was washed successively with pre-cooled acetone (−20 °C) and dissolved in 3 mL of 1 mM sulfuric acid. The resulting solution was measured for absorbance at 410 nm.

O_2_^−^ Content measurement: In 4 mL of 65 mM phosphate buffer solution (PBS, pH = 7.8), 0.2 g of sample was added and grinded well and then centrifuged (4 °C, 10,000 rpm, 15 min). Then, 0.5 mL of the supernatant was taken and mixed with 1 mL of hydroxylamine hydrochloride and 0.5 mL of 65 mM phosphate buffer and left for 1 h. One mL of p-aminobenzenesulfonamide (17 mM) and one mL of α-naphthylamine (7 mM) were added to the above mixture and incubated at 25 °C for 20 min. An equal volume of ether was added and centrifuged (4500 rpm, 3 min), the supernatant was taken, and the absorbance value was measured at 530 nm.

### 4.9. Measurement of Proline Content

Proline was determined using the ninhydrin colorimetric method [82]. Into 0.5 g of sample, 2.5 mL of glacial acetic acid was added and centrifuged (10,000 rpm for 2 min) after 10 min in a boiling water bath. One mL of the supernatant was reacted with two mL of acidic ninhydrin solution for 30 min in a boiling water bath and then centrifuged (10,000 rpm for 2 min) and finally extracted by adding toluene. The absorbance at 520 nm was measured by UV spectrophotometer and calculated.

### 4.10. Measurement of Endogenous Hormone Content

Maize seeds of CK, S, and SM50 were taken at 19 h, 36 h, and 60 h of germination, respectively, and endogenous hormone contents were determined. The milled samples were mixed with methanol/water/formic acid (15:4:1, *v*/*v*/*v*). The mixture was shaken for 10 min and then centrifuged (4 °C, 12,000 rpm, 5 min). The supernatant was evaporated and dissolved in 80% methanol (*v*/*v*), then filtered through a membrane filter and further detected using high-performance liquid chromatography/mass spectrometry (HPLC-MS, column: Waters ACQUITY UPLC HSS T3 C18, 1.8 μm, 2.1 mm × 100 mm; solvent system: water with 0.04% acetic acid: acetonitrile with 0.04% acetic acid; gradient program: 90:10 *v*/*v* at 0 min, 40:60 *v*/*v* at 5.0 min, 40:60 *v*/*v* at 7.0 min, 90:10 *v*/*v* at 7 min and 90:10 *v*/*v* at 10 min; injection volume: 2 μL). The contents of IAA, GA_3_, JA, and ABA in the seeds were determined by Wuhan Metware Biotechnology Co. (Wuhan, China). Each treatment contained three biological replicates.

### 4.11. Transcriptome Sequencing and Analysis

Total RNA was isolated from maize seeds at 19 h, 36 h, and 60 h from the S and SM50 treatment groups using RNA isolater Total RNA Extraction Reagent produced by Nanjing Novozymes Biotechnology Co., Ltd. (Nanjing, China) according to the instruction manual. Each treatment contained three biological replicates. Transcriptome sequencing was entrusted to Beijing Novozymes Technology Co. (Beijing, China) using the Illumina sequencing platform. Company-delivered Clean Data were aligned to the maize B73 reference genome (Zm-B73-REFERENCE-NAM-5.0) using Hisat2 (v2.2.1), followed by quantification of gene expression using featureCounts (v2.0.0). DEGs were screened between SM50 treatment and S treatment using DESeq2 (v1.38.3). GO enrichment analysis and KEGG enrichment analysis of DEGs and gene set enrichment analysis (GSEA) of all genes in each treatment separately were performed using clusterProfiler (v4.6.1). Time series analysis of DEGs was based on soft clustering-based Mfuzz in CluserGVis (v0.0.4).

### 4.12. Quantitative Real-Time PCR Assays

Quantitative real-time PCR (qRT-PCR) assays were performed using the same RNA samples as used for transcriptome analysis, with each sample containing three biological replicates. The gene-specific primers used for qRT-PCR assays are listed in Table S1. qRT-PCR was performed with SYBR Green Premix Pro Taq HS Kit II (Accurate Biotechnology, Changsha, China) on a LightCycler^®^ 96 Instrument fluorescent quantitative PCR instrument. Relative expression was calculated by the 2^−∆∆CT^ method [83].

### 4.13. Data Processing and Analysis

One-way ANOVA was used to assess the significance of differences and the final results were expressed as mean ± standard deviation. Data were statistically and analyzed using SPSS 22.0, and *p* < 0.05 was considered a statistically significant difference. Downstream analysis of transcriptome data and graphical plotting were performed in R (v4.2.2).

## 5. Conclusions

In conclusion, salt stress inhibited seed germination, but MT pretreatment promoted maize seed germination. Melatonin pretreatment alleviated the inhibitory effect of salt stress on maize seed germination by increasing the antioxidant ability of maize seeds under salt stress and reducing the accumulation of ROS, as well as the content of MDA. Meanwhile, melatonin was involved in regulating the content changes of endogenous hormones during the germination of maize seeds under salt stress, which reduced the content of ABA and JA, increased the content of IAA and GA_3_, and broke the balance between GA_3_ and ABA to promote the germination. Melatonin also regulated the expression of genes related to starch and sucrose metabolism pathways and phytohormone signal transduction pathways by affecting the expression of antioxidant enzymes, response to stress, and seed germination-related genes in maize seeds under salt stress and affected the expression of different functional genes at different germination stages, thus achieving the enhancement of seed salt tolerance throughout the germination stage.

## Data Availability

The RNA-seq dataset in this study has been uploaded to SRA database in NCBI (BioProject ID: PRJNA1120961).

## Supplementary Materials

The following supporting information can be downloaded at: https://www.mdpi.com/article/10.3390/plants13152142/s1, Table S1: Primers designed by qRT-PCR; Table S2: Transcriptome sequencing quality assessment; Figure S1: The phenotypic differences in seed morphology at 5th day. Genes: Zm00001eb283530, Zm00001eb003440, Zm00001eb148130 and Zm00001eb327450. SM: germination of seeds treated with 50 μM melatonin under 150 mM NaCl solution; S: germination of seeds treated with 150 mM NaCl. Figure S2: Effects of melatonin on gene expression under salt stress in maize seed by qRT-PCR.
