# Supplementary material for: The Physiological and Molecular Mechanisms of Exogenous Melatonin Promote the Seed Germination of Maize (Zea mays L.) under Salt Stress
Source: Plants (Basel). 2024 Aug 2;13(15):2142. doi: 10.3390/plants13152142 (PMC11313997; doi:10.3390/plants13152142)
Supplement: Supplementary file 1 [file plants-13-02142-s001.zip › plants-3081347-supplementary.pdf]

Table S1 Primers designed by qRT-PCR

GeneID	Description	F	R
ZmActin	actin	GCATCCATGAGACCACCTACAAC	GATGGACCCTCCTATCCAGACAC
Zm00001eb003440	BAM	CTGGCAGGAGATGGGTTTGA	GACACCTTCTGACCGCTGAA
Zm00001eb283530	HK	CAGTGCCATACCCAAGCTCA	TACATGCCCGAAACCAGCTT
Zm00001eb148130	PP2C1	TTCCATTGCACCCATTTCCG	GCAAGTGTCTGTCTCCCACA
Zm00001eb327450	JAZ1	GGGGTCCTTTATAAGCGCGT	CAGCCGGGCAAGCAAATATC

Table S2 Transcriptome sequencing quality assessment

Sample	Raw Reads	Clean Reads	Q20(%)	Q30(%)	GC(%)
S-19h-1	50170582	48492662	97.14	92.61	54.17
S-19h-2	56216830	53871318	97.05	92.53	54.94
S-19h-3	49869260	47863478	97.13	92.65	55.35
S-36h-1	44408086	42363866	97.03	92.43	54.88
S-36h-2	47592900	46162992	97.11	92.56	54.5
S-36h-3	40916986	39843876	96.95	92.16	54.6
S-60h-1	44042048	42111130	97.24	92.86	54.52
S-60h-2	45799218	44002242	97.05	92.5	55.75
S-60h-3	51365944	49075924	96.88	92.2	56.51
SM50-19h-1	50563158	48355698	96.83	91.99	55.25
SM50-19h-2	46025236	44630870	97.02	92.46	55.6
SM50-19h-3	49671198	47257758	97.06	92.56	55.35
SM50-36h-1	44086684	41525156	97.38	93.18	53.23
SM50-36h-2	54919890	53230878	97.16	92.65	54.35
SM50-36h-3	57897858	55627220	97.27	92.94	54.22
SM50-60h-1	53970370	52338292	97.01	92.48	56.15
SM50-60h-2	46635174	45338022	96.53	91.22	56.48
SM50-60h-3	51089748	49311258	96.84	92.08	56.29

Note: Sample: sample name; Raw Reads: number of unfiltered raw reads; Clean Reads: number of filtered high quality reads; Q20: proportion of Qphred values greater than 20; Q30: proportion of Qphred values greater than 30; GC: ratio of GC base content in Clean Reads.

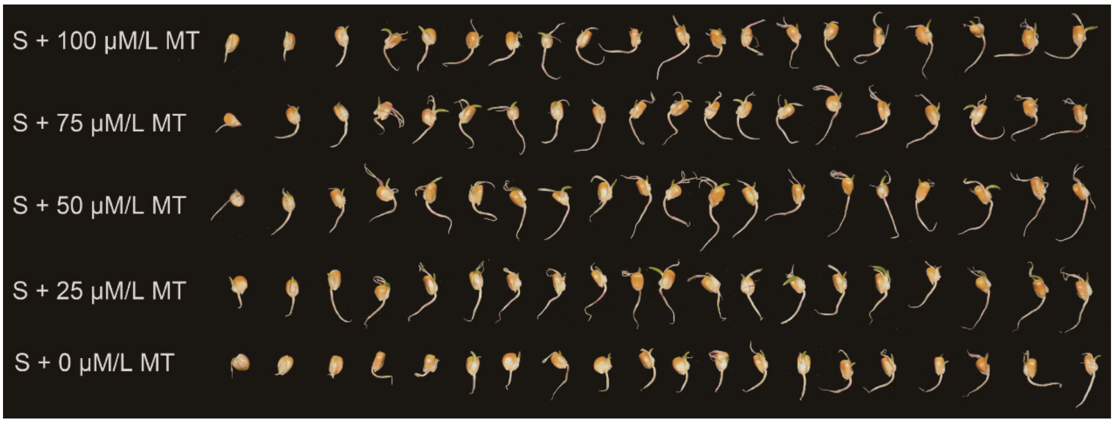

Figure S1. The phenotypic differences in seed morphology at 5th day.

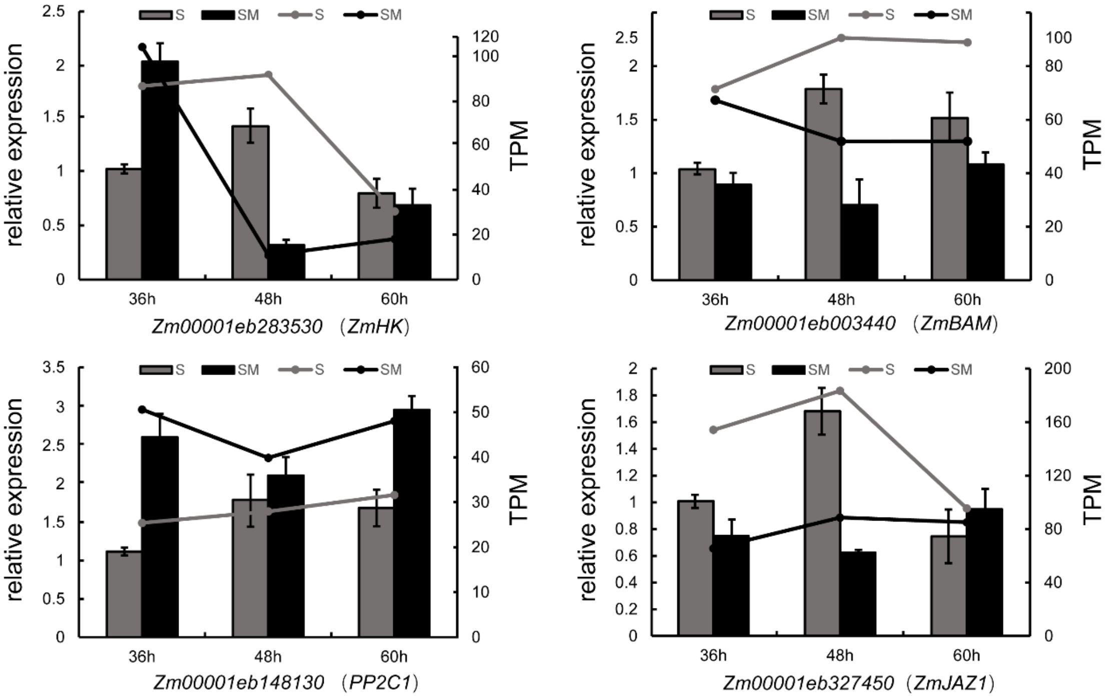

Figure S2. Effects of melatonin on gene expression under salt stress in maize seed by qRT-PCR. Genes: Zm00001eb283530, Zm00001eb003440, Zm00001eb148130 and Zm00001eb327450. SM: germination of seeds treated with 50 μM melatonin under 150 mM NaCl solution; S: germination of seeds treated with 150 mM NaCl.
